# Red Blood Cell Distribution Width to Platelet Count Ratio Facilitates Preoperative Prediction of Recurrence in Surgically Treated Chronic Subdural Hematoma

**DOI:** 10.3389/fneur.2022.884231

**Published:** 2022-05-11

**Authors:** Ági Güresir, Christoph Coch, Annkristin Heine, Elvira Mass, Tim Lampmann, Hartmut Vatter, Markus Velten, Marie-Therese Schmitz, Erdem Güresir, Johannes Wach

**Affiliations:** ^1^Department of Neurosurgery, University Hospital Bonn, Bonn, Germany; ^2^Department of Clinical Chemistry and Clinical Pharmacology, University Hospital Bonn, Bonn, Germany; ^3^Department of Internal Medicine III for Hematology, Oncology, Rhemuatology and Immune-Oncology, University Hospital Bonn, Bonn, Germany; ^4^Life and Medical Sciences Institute (LIMES), Developmental Biology of the Immune System, University of Bonn, Bonn, Germany; ^5^Department of Anesthesiology and Critical Care Medicine, University Hospital Bonn, Bonn, Germany; ^6^Department of Medical Biometry, Informatics and Epidemiology, Medical Faculty Rheinische Friedrich-Wilhelms-University Bonn, Bonn, Germany

**Keywords:** chronic subdural hematoma, inflammation, platelet count, red blood cell distribution width, recurrence

## Abstract

**Objective:**

Recent studies have demonstrated emerging evidence of the role of inflammation in the growth and recurrence of chronic subdural hematoma (cSDH). Red blood cell distribution width to platelet count ratio (RPR) is a novel biomarker for inflammation in cancer, cardiac, and inflammatory diseases. The present retrospective study investigated the impact of RPR on recurrence after burr hole surgery for cSDH in 297 patients

**Methods:**

The optimal cut-off value for RPR was defined as ≥0.0568 according to the receiver operating characteristic curve (AUC:0.64, 95%CI:0.55–0.72, *p* = 0.007). The study cohort was dichotomized into low (*n* = 157) and high (*n* = 140) RPR groups

**Results:**

Significant differences between the groups were identified regarding American Society of Anesthesiologists (ASA) classification and frequency of anticoagulant intake. Demographics, comorbidities, size, morphology, and mass effect of cSDH were homogeneously distributed among the RPR groups. Multivariable binary logistic regression analysis considering location, midline-shift, septation, RPR, anticoagulant intake, sex, and ASA classification revealed that an increased baseline RPR (≥0.0568, OR: 3.1, 95%CI: 1.4–6.8, *p* = 0.004), and preoperative midline-shift (≥5 mm, OR: 2.7, 95%CI: 1.3–6.0, *p* = 0.01) are independent predictors of recurrent cSDH.

**Conclusion:**

The present findings suggest RPR as a novel inflammatory biomarker enabling risk stratification of recurrence after burr hole surgery for cSDH and might facilitate tailored medical decision making.

## Graphical Abstract

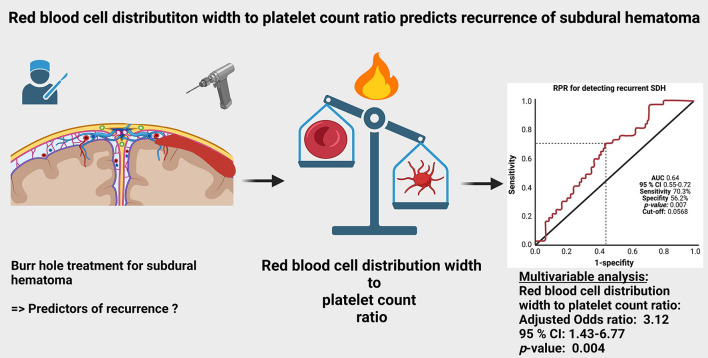

## Introduction

Chronic subdural hematoma (cSDH) is a widespread neurological condition, especially in elderly patients. Due to the global population aging, it is of paramount importance to make reliable forecasts regarding the outcomes and treatment options. To date, surgery is predominantly considered the mainstay of therapy (1–6).

There is emerging evidence that traumatic injury and hemorrhage are not the only sources of growth and development of subdural hematomas (7, 8). Several recent studies (9–12), including Virchow (13), have suggested that inflammatory burden is also one of the main avenues in the development of a cSDH.

There is an increasing amount of data emphasizing the prognostic value of systemic inflammatory laboratory values. However, values such as c-reactive protein (CRP) or white blood cell (WBC) count have commonly used parameters that are often influenced by several comorbidities or widespread drugs such as corticosteroids. Red blood cell distribution width (RDW) has been found to be a prognostic variable in several conditions such as cancer, traumatic brain injury, and cardiac diseases (14–17). Furthermore, emerging evidence recommends the use of RDW to platelet count ratio (RPR) regarding the reflection of systemic inflammation. RDW and platelet count are easy and quick-to-use preoperative laboratory parameters that might facilitate a tailored prediction of prognosis and comprehensive consultation with patients and their relatives regarding medical decision making. To date, the RPR has not yet been investigated in its predictive value regarding the recurrence of cSDH.

Therefore, the present study investigates patients who underwent single burr hole craniostomy for cSDH regarding the risk stratification of recurrent SDH focusing on the use of the RPR.

## Methods

### Study Design

A total of 297 patients who underwent neurosurgical treatment via burr-hole craniostomy for chronic subdural hematoma were retrospectively reviewed. The present investigation aimed to analyze clinical data regarding demographics (age, sex), medical history (hypertension, diabetes mellitus, heart disease, chronic renal failure), anticoagulant or antiaggregant intake, history, and time from trauma to surgery, recurrence, and functional outcome. Computed tomography (CT) scans were used as confirmation of the presence of cSDH. Both pre- and postoperative CT scans were collected from the medical imaging platform XERO Universal Viewer (Agfa HealthCare, Gent, Belgium). CT scans were reviewed regarding location (uni- or bilateral), hematoma size (preoperative width of hematoma in axial scan), septation within the hematoma, and extent of midline-shift. Midline-shift was measured in millimeters, as the perpendicular distance between the ideal midline and the septum pellucidum. The line being coplanar with the falx cerebri was considered the ideal midline (18–20). Preoperative midline-shift was dichotomized according to the median-split method (≥5/ <5 mm). Concerning laboratory examinations, values such as baseline serum CRP, hemoglobin (Hb), WBC count, platelet count, and RDW were determined in the routinely sampled preoperative laboratory investigations obtained at the time of hospital admission and measured as described previously (21, 22).

### Surgical Workflow

Preoperatively, the subgroup of patients who were treated with anti-coagulants underwent a normalization of their coagulation status using vitamin k, fresh frozen plasma, or single vitamin-k-dependent factors such as factor VII. Patients taking antiplatelet drugs were instructed to discontinue intake immediately at the time of diagnosis, and surgical evacuation was performed within 48 h depending on the degree of the patient's functional status. The neurosurgical subdural hematoma evacuation was performed under general anesthesia. The surgical treatment was performed as single-burr-hole craniostomy. Repeated irrigation using physiological saline solution was performed to evacuate the subdural hematoma. Afterward, a closed-system subdural drain insertion was performed, and those drains usually remained *in situ* for 2–3 days. The time to removal of the drain depended on the amount of subdural fluid collection and the reduction of the mass effect determined by the postoperative CT scan performed within 72 h after surgery (6).

### Outcome Evaluation

Neurological function was graded using the Karnofsky performance status (KPS) scale at discharge and at follow-up examinations (23). All patients were routinely examined using clinical and imaging follow-up by CT scans at our outpatient clinic 14 days after discharge. In case of residual or persistent subdural hematoma, patients were kept for further follow-up appointments until sufficient resorption of the subdural hematoma. Recurrent cSDH was defined as an increase of the subdural hematoma volume with compression of the brain surface compared to CT scans prior to and after hematoma evacuation in combination with the onset of new or progressive neurological deficits (6).

### Statistics

Data were organized and analyzed using SPSS for Windows (v27.0; IBM Crop, Armonk, NY, USA). All values were expressed as mean ± SD unless otherwise stated. Receiver-operating characteristic (ROC) curves were constructed and the area under the receiver-operating characteristic curve (AUC) for RPR in the prediction of recurrent cSDH was determined. A frequency distribution histogram of RPR values was created. Binomial group testing included the presentation of 95% confidence intervals based on the Clopper–Pearons method and was used to calculate the power size level. Analyses regarding power size level calculation were performed using the R software v4.04 (R Foundation for Statistical Computing, Vienna, Austria). Power size level calculations based on the observed recurrence rates were performed by assuming to achieve a power level of 0.80 and a significance level of 0.05. Power level calculation revealed that at least 130 patients in each arm are necessary to achieve the mentioned criteria. Univariable analyses of the proportions of categorical data among the RPR groups were performed using Pearsons's chi-squared test (two-sided). A *p*-value < 0.05 was defined as statistically significant. Moreover, a multivariable binary logistic regression analysis was performed to analyze independent predictors of recurrent cSDH.

## Results

### Patient Characteristics

A total of 297 consecutive patients with cSDH were analyzed. Mean age was 77.5 ± 9.1 years with a male predominance (female: male = 1:1.74). 220 (220/297; 74.1%) patients suffered from unilateral, and 77 (77/297; 25.9%) patients from bilateral cSDH. Midline-shift at diagnosis was 4.9 ± 4.4 mm, and axial diameter was 10.0 ± 9.3 mm. Intraoperative or radiological septation of the cSDH was observed in 101 (34.0%) cases. The time from trauma to surgery was 27.6 ± 20.3 days.

### Red Blood Cell Distribution Width to Platelet Count Ratio and Cut-Off Determination

**Figure 1:**
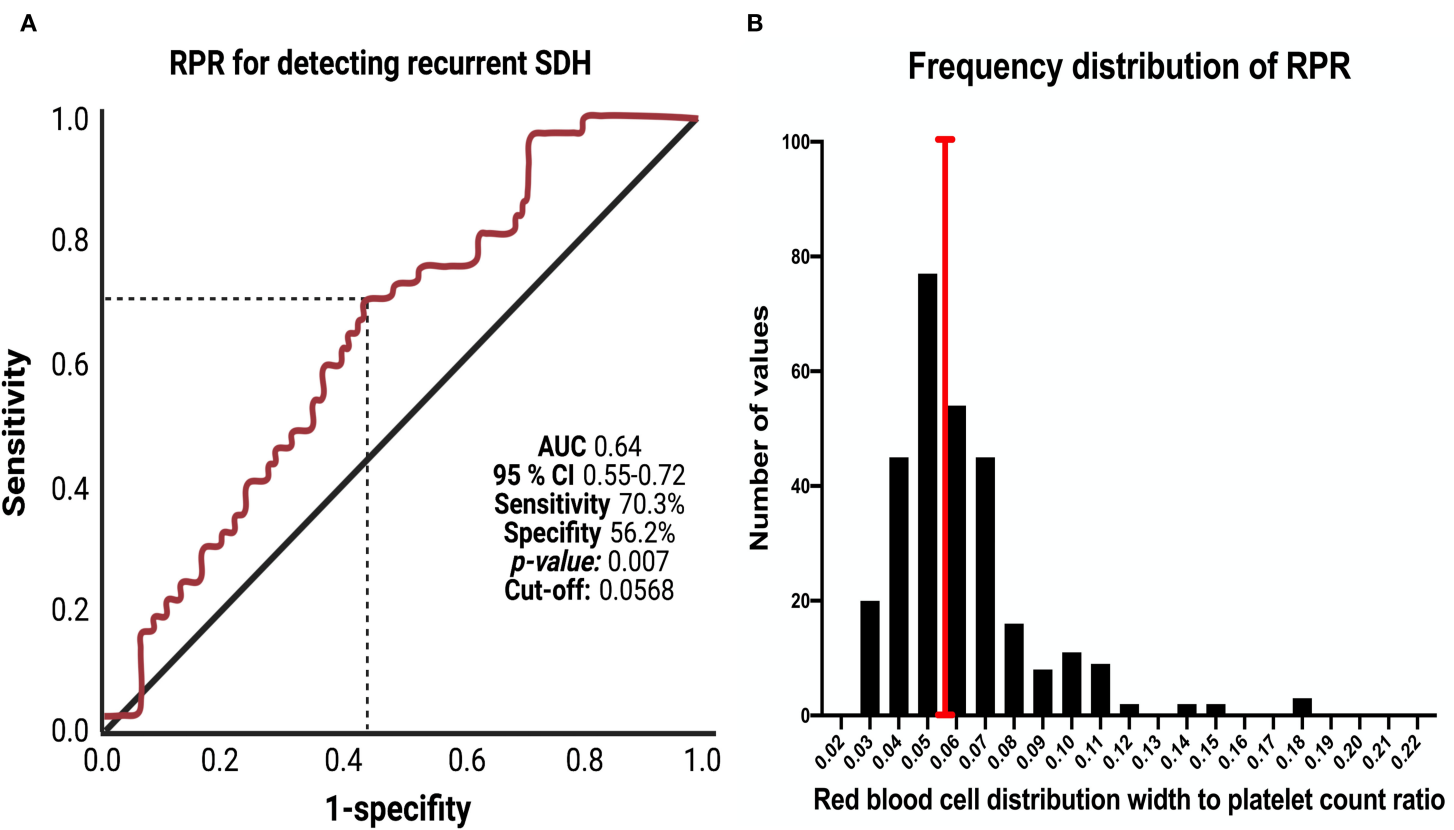
**(A)** Receiver-operating characteristic curve illustrating the ability of baseline RPR to predict recurrence of cSDH. The area under the ROC curve (AUC) of baseline RPR for recurrent cSDH was 0.64 (95% confidence interval (CI): 0.55–0.72). Sensitivity and specificity of baseline RPR for predicting recurrent cSDH were 70.3% and 56.2%, respectively, with a threshold of ≥ 0.0568. **(B)** Frequency distribution histogram for baseline RPR in the investigated cohort. The black bars indicate the number of patients with the corresponding RPR values. The red vertical line displays the optimized cut-off point for baseline RPR.

The mean RPR was 0.0647 ± 0.0379. A ROC curve was constructed to determine a RPR cut-off value in the prediction of recurrent cSDH. The AUC of baseline RPR for recurrent cSDH was 0.64 (95% Confidence interval (CI): 0.55–0.72, *p* = 0.007; Figure 1A). The sensitivity and specificity of baseline RPR for predicting recurrent cSDH were 70.3 and 56.2%, respectively, with a threshold of ≥0.0568. Figure 1B displays the optimal cut-point (red line) and the corresponding frequency distribution histogram for baseline RPR values in the cohort.

### Patient Characteristics of RPR Groups

The study cohort was dichotomized into patients with cSDH with low (<0.0568) and high (≥0.0568) baseline RPR values. A total of 157 (157/297; 52.9%) patients were allocated to the “low” RPR group and 140 (47.1%) to the “high” RPR group. Univariable analyses using Pearson's chi-squared test (two-sided) and independent *t*-test were performed regarding the distribution of demographics, comorbidities, imaging characteristics of cSDH, medication, serum CRP and hemoglobin. American Society of Anesthesiologists physical status classification system (ASA) was the only heterogeneously distributed variable among the RPR groups. The intake of antiplatelet or anticoagulants only or the combination of both was not heterogeneously distributed among the RPR groups. Mean (±) RPR in patients who took antiplatelets was 0.064 (± 0.02), whereas patients without prescribed antiplatelets had a mean RPR of 0.069 (±0.06; independent *t-*test: *p* = 0.69). Eighty-five (60.7%) patients had an ASA score >2 in the high RPR group, and 70 (44.6%) patients had an ASA score >2 in the low RPR group, respectively (*p* = 0.005). 97 (97/140; 69.3%) patients took blood thinners (only anticoagulants or antiplatelet drugs & combination of anticoagulants and antiplatelets) in the high RPR group, whereas 87 (87/157; 55.4%) patients took those drugs in the low RPR group (*p* = 0.01). Further details are summarized in Table 1.

**Table 1:** Comparison of low- vs. high-Red blood cell distribution width / Platelet Count ratio group (using Pearson's chi-squared test (two-sided) and independent *t*-test).

**Characteristics**	**Low-RPR (157/297; 52.9%)**	**High-RPR (140/297; 47.1%)**	***p*-Value**
Age (years), mean ± SD	76.8 ± 9.7	78.2 ± 8.4	0.19
Sex			0.02
Female	66 (42.0%)	41 (29.3%)	
Male	91 (58.0%)	99 (70.7%)	
ASA PS ≤ 2 >2	87 (55.4%) 70 (44.6%)	55 (39.3%) 85 (60.7%)	0.005
Location Unilateral Bilateral	114 (72.6%) 43 (27.4%)	104 (74.3%) 36 (25.7%)	0.74
Preoperative midline-shift (mm), mean ± SD	5.0 ± 4.3	4.8 ± 4.6	0.69
Preoperative axial SDH size, mean ± SD	10.2 ± 8.6	9.7 ± 10.0	0.69
Septated SDH Present Not present	52 (33.1%) 105 (66.9%)	49 (35.0%) 91 (65.0%)	0.73
Time from trauma to surgery (d), mean ± SD	38.8 ± 25.2	36.5 ± 24.4	0.50
Arterial hypertension Present Not present	100 (63.7%) 57 (36.3%)	77 (55.0%) 63 (45.0%)	0.13
Anticoagulant intake yes no	61 (38.9%) 96 (61.1%)	59 (42.1%) 81 (57.9%)	0.65
Antiplatelet intake yes no	11 (7.0%) 146 (93.0%)	14 (10%) 126 (90.0%)	0.47
Anticoagulant + Antiplatelet intake yes no	15 (9.6%) 142 (90.4%)	24 (17.1%) 116 (82.9%%)	0.08
Diabetes mellitus Present Not present	28 (16.8%) 129 (82.3%)	32 (22.9%) 108 (77.1%)	0.87
Chronic renal failure Present Not present	17 (10.8%) 140 (89.2%)	9 (6.4%) 131 (93.6%)	0.18
Baseline serum CRP	16.5 ± 25.6	13.2 ± 25.1	0.27
Baseline White blood cell count	9.0 ± 2.5	8.6 ± 2.3	0.12
Baseline hemoglobin	13.0 ± 1.8	13.0 ± 2.2	0.87

*ASA PS, American society of anesthesiologists physical status classification system; CRP, C-reactive protein RPR, Red blood cell distribution width to platelet count ratio; SD, Standard deviation; SDH, Subdural hematoma*.

### RPR in the Prediction of Recurrent cSDH

37 (37/297, 12.5%, 95% CI: 8.9–16.8) patients with a recurrent cSDH were identified in the entire cohort. Eleven (11/157; 7.0%, 95% CI: 3.6–12.2) patients with recurrent cSDH were in the low RPR group, whereas 26 (26/140; 18.6%, 95% CI: 12.5–26.0) patients were in the high RPR group (*p* = 0.003). The mean time to recurrence was 29.3 ± 22.5 days in the low RPR group, and 26.9 ± 19.6 in the high RPR group, respectively (*p* = 0.75).

**Figure 2:**
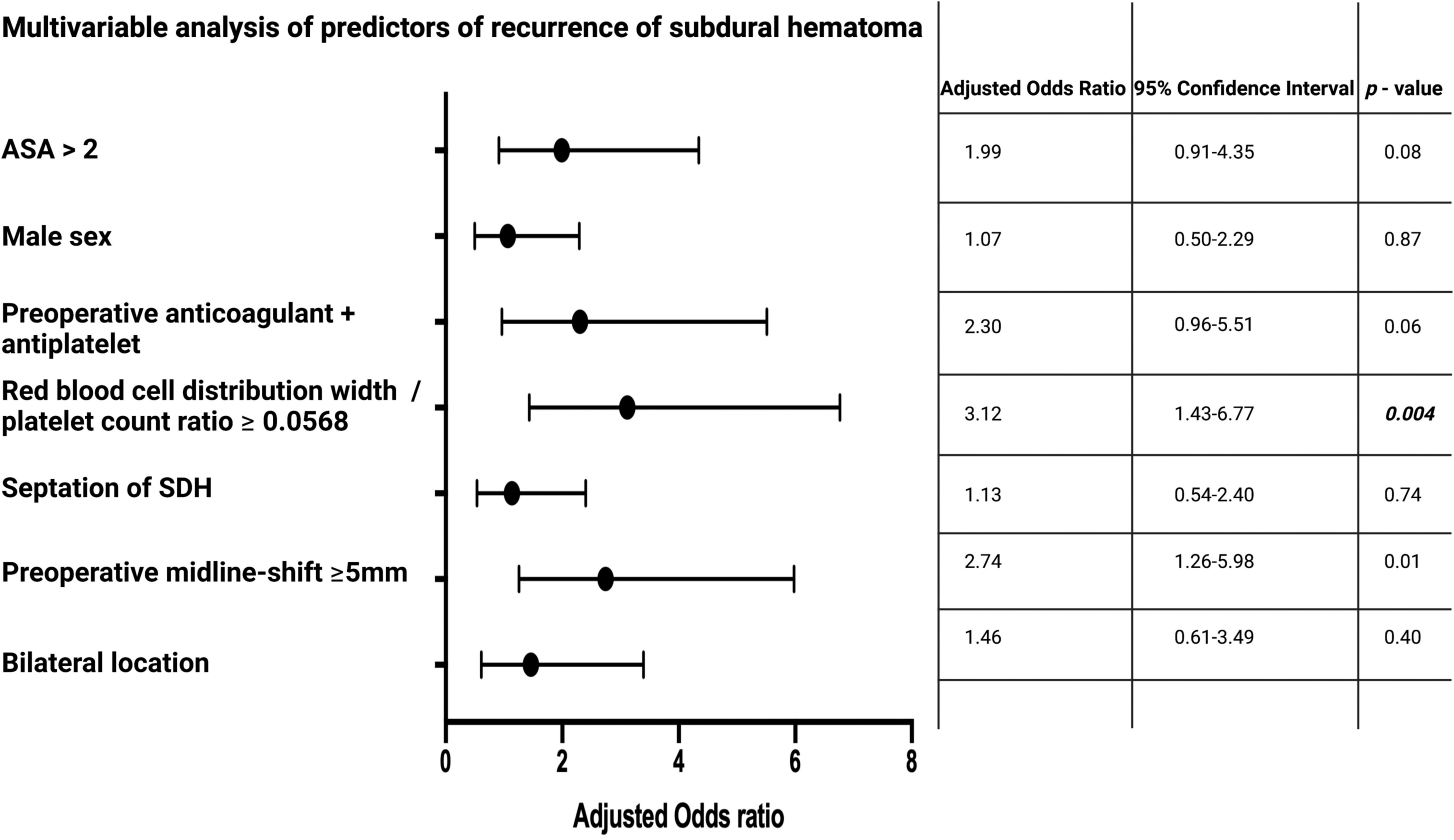
Forest plots from multivariable logistic regression analysis: High red blood cell distribution width to platelet count ratio (≥0.0568), and a preoperative midline-shift ≥5 mm are independent predictors of cSDH recurrence. *p*-Values in italics and bold display statistically significant results.

Multivariable binary logistic regression analysis of recurrent cSDH considering sex (male/female), location (bilateral/unilateral), preoperative midline-shift (≥5/ <5 mm), septation of cSDH (present/not present), RPR (≥0.0568/ <0.0568), preoperative anticoagulant or antiplatelet intake (yes/no), and ASA score (>2/ ≤ 2) was performed. The multivariable analysis identified high RPR (≥0.0568) (adjusted odds ratio (OR): 3.12, 95% CI: 1.43–6.77, *p* = 0.004), and preoperative midline-shift ≥ 5 mm (OR: 2.74, 95% CI: 1.26–5.98, *p* = 0.01) as significant and independent predictors of cSDH recurrence. Figure 2 summarizes the results of the multivariable analysis.

### Septation of Chronic Subdural Hematoma and Inflammatory Markers

Septations of chronic subdural hematomas on CT scans were observed in 101 (101/297; 34.0%). An analysis of the association between laboratory inflammatory markers and the presence of septations was performed. Patients with a septated cSDH had a mean (±) baseline RPR of 0.069 (± 0.05), whereas patients with cSDH and without septation had a mean baseline RPR of 0.062 (± 0.03; independent *t*-test: *p* = 0.18). Further analyses were performed regarding the associations of baseline serum CRP and WBC count in patients with or without septated cSDHs. The results of those analyses are summarized in Table 2.

**Table 2:** Comparison of baseline inflammatory markers in patients with or without septated chronic subdural hematomas (using independent *t*-test).

**Variable**	**Mean ±SD**	**Mean difference**	**95% CI of the Difference**	***p*-value**
Baseline Red blood cell distribution width to platelet count ratio Septated cSDH Non-septated cSDH	0.069 ± 0.05 0.062 ± 0.03	−0.007	−0.017–0.003	0.175
Baseline serum C-reactive protein Septated cSDH Non-septated cSDH	19.1 ± 30.5 12.7 ± 21.9	−6.4	−13.1–0.42	0.066
Baseline white blood cell count Septated cSDH Non-septated cSDH	9.1 ± 2.5 8.6 ± 2.4	−0.45	−1.03–0.13	0.13

### Clinical Outcome

**Figure 3:**
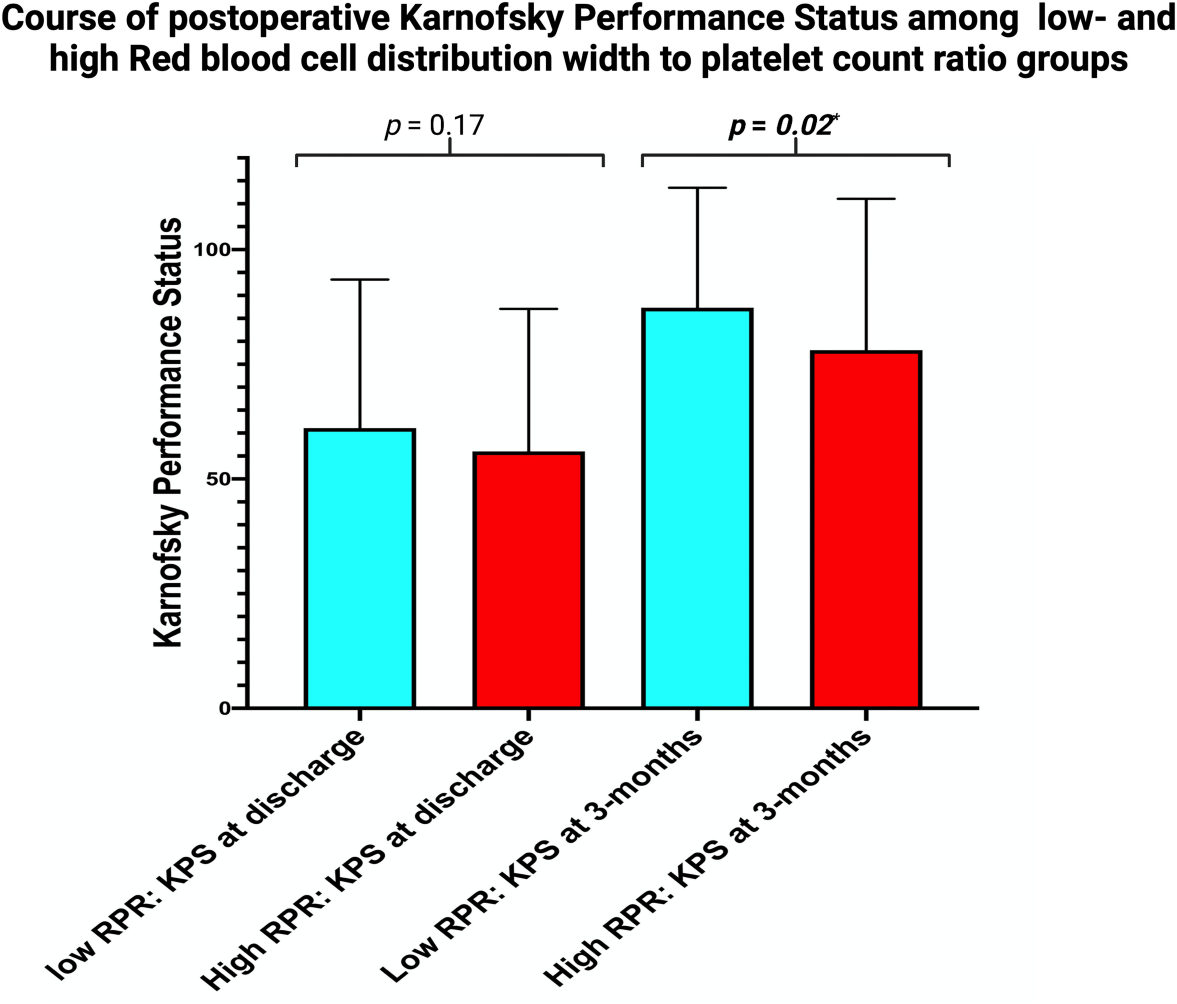
Column bars displaying the mean value of KPS stratified by the parameters “low RPR” (light blue) and “high RPR” (red) at discharge and after 3 months. The whiskers represent the standard deviation. *p*-Values of the paired *t*-test comparing the mean values are shown.

KPSs at discharge were homogeneously distributed among the RPR groups. Mean KPS at discharge was 61.1 ± 32.4 in the low RPR group, and 56.0 ± 31.1 in the high RPR group (*p* = 0.17). After 3 months, KPSs improved in both groups but was significantly higher in the low RPR (87.4 ± 26.2) compared to the high RPR group (78.1 ± 33.0, *p* = 0.02) (Figure 3).

## Discussion

The present investigation reveals the red blood cell distribution width-to-platelet count ratio (RPR) as a simple and quick-to-use preoperative laboratory marker which is an independent predictor of cSDH recurrence and might facilitate medical decision making.

To date, the main avenue of therapy for cSDH is surgical evacuation and the insertion of a subdural drain. Recurrence rates after surgical evacuation of a chronic subdural hematoma range from 3 to 39%, and might necessitate repeat surgery (4, 24, 25). Therefore, a comprehensive and tailored preoperative assessment of the risk/benefit ratio and prognosis is of paramount importance prior to therapy for the predominantly elderly patients. Various theories involving angiogenesis, fibrinolysis, and inflammation exist regarding cSDH formation and the development of a recurrent cSDH (9). Kalamatianos et al. (10) quantified the levels of placental growth factor (PlGF) and soluble vascular endothelial growth factor (sVEGFR-1) in the serum and hematoma fluid. PlGF and sVEGFR-1 were significantly higher in hematoma fluid compared to serum. Several other studies confirmed this interesting finding and revealed higher levels of VEGF and VEGF-R in cSDH fluid compared to peripheral blood and cerebrospinal fluid (26–31). The origin of VEGF in the hematoma is highly debated and several studies assumed that it might be produced by neutrophils within the cSDH fluid, vascular endothelial cells of the cSDH membrane or macrophage infiltrates (27–29, 32). Those findings were also correlated with clinical endpoints. For instance, a prospective trial investigating the subdural fluid concentrations of VEGF, bFGF, and interleukin (IL)-6 in recurrent and non-recurrent cSDHs revealed that the concentrations of IL-6 were significantly higher in the group of recurrent cSDH patients. Furthermore, they found that the immunohistochemical staining of VEGF was significantly stronger in recurrent cSDH patients (33). Therefore, higher levels of VEGF expression in the outer membrane and increased inflammatory burden in the cSDH fluid have been associated with a higher probability of recurrence in cSDH patients. The internal membrane of cSDH was shown to be predominantly consisting of collagen and fibroblasts, whereas consists of fibroblasts, collagen fibers, neurtrophils, lymphocytes, macrophages, and eosinophils. The external membrane is considered as the origin of driving growth of cSDH (34–39). However, the additional fenestration of the internal cSDH membrane is also highly debated regarding the recurrence and recent data revealed lower recurrence rates compared to surgical evacuation without fenestration of the internal membrane (40, 41). VEGF expression is regulated by inflammatory mechanisms such as Prostaglandin E (PGE2), which is synthesized from arachidonic acid by cyclooxygenase (COX)-2 (42). Moreover, PGE2 levels were demonstrated to be correlated with the time interval between trauma and cSDH diagnosis.

Recently, RDW and RPR gained increasing attention as biomarkers of inflammation, and predictors of outcomes in cardiac, cancer, and infectious diseases (43–47). RPR is recognized as a valuable predictive indicator of systemic inflammatory response. However, the specific mechanism elucidating the poor prognosis regarding recurrence of cSDH in patients with elevated RPR remains predominantly unexplained. With the recurrence of cSDH, an extensive inflammatory reaction might be triggered and result in an increase of the levels of cytokines such as interleukin-6, tumor necrosis factor-α, and hepcidin (48, 49). Those cytokines might downregulate the maturation of erythrocytes and expedite the entry of new and larger reticulocytes into the peripheral blood circulation, which results in an increase in the RDW (50). Furthermore, platelets are also known to release several growth factors such as platelet-derived growth factor, VEGF, and platelet factor 4, which might drive the regrowth of cSDH (51). Nevertheless, the pathophysiological imbalance between RDW and platelet count in a ratio functioning as a predictor of growth and recurrence of cSDH remains to be further investigated.

The baseline determination of RPR seems to be useful to identify a subgroup of patients suffering from an increased inflammatory burden who might be predisposed to recurrence of cSDH which also results in a poor course of postsurgical KPS. There are some potential clinical implications for future trials and the clinical care of those predominantly elderly and vulnerable patients. Steroids have long been suggested as a treatment option for cSDH (52, 53). Dexamethasone was found to induce the formation of significantly smaller and lighter blood clots in cSDH. Moreover, dexamethasone might inhibit the inflammatory response and the development of membranes, which is of paramount importance regarding repeated hemorrhages and cSDH growth (54). Several small size studies have demonstrated that dexamethasone could reduce the recurrence of cSDH following surgery in patients who underwent a perioperative dexamethasone therapy compared to patients who underwent surgery only (55, 56). Furthermore, there is also prospective data supporting the use of dexamethasone in nonsurgical treatment for cSDH (57). Dexamethasone is known to be an anti-inflammatory drug, which alters the gene expression and transcription of inflammatory mediators such as cytokines and chemokines (58–60). Moreover, dexamethasone affects the differentiation and polarization of several immune cells, such as lymphocytes, and macrophages (61). Steroids such as dexamethasone are also capable to influence vascular permeability and reduce the permeability of the blood-brain-barrier permeability by inducing the expression of the gene occludin which modifies the endothelium and the tight junctions (62). Those mechanisms might result in a reduction of the blood-brain-barrier permeability and attenuates the accumulation of fluid, and immune cells which drive the inflammation such as leukocytes. Hence, it was also suggested that dexamethasone acts in a similar way on the vascular endothelial cells of the vulnerable blood vessels observed in membranes of cSDH by reducing the exudation of hematoma and enabling resolution of the fluid (9). However, the definitive pathophysiological mechanism of dexamethasone in cSDH remains unclear yet. A major limitation of a general recommendation for the use of dexamethasone in all patients with cSDH might be the side effect profile such as diabetes, increased risk of intestinal bleedings, and ulcers, which potentially affects the predominantly elderly patients suffering from cSDHs (63, 64). However, Dexamethasone failed to improve outcomes in a prospective randomized trial (65). RPR might be a simple and quick-to-use biomarker to identify a subgroup of cSDH patients who have an increased inflammatory burden and increased risk of recurrence. Hence, this potential quick-to-use biomarker can facilitate a comprehensive preoperative consultation with elderly patients and their relatives. This biomarker could be of potential interest for those patients without clinical and imaging “red flags” requiring surgical evacuation. Increased baseline RPR might facilitate the identification of patients who will benefit from potential conservative drug therapy options with an anti-inflammatory approach. In addition to the emerging data about dexamethasone in cSDH, the use of atorvastatin in patients with cSDH is also increasingly debated. For instance, a randomized controlled trial enrolled patients with cSDH and they were treated with either atorvastatin or placebos. Patients who received atorvastatin had a significantly more regredient hematoma volume on CT-imaging and improved significantly more regarding neurological functioning compared to the placebo arm (66). This conclusion regarding the potential benefit of atorvastatin via suppression of the inflammatory burden was also reconfirmed by the results of a recently pooled meta-analysis investigating six trials concerning the use of atorvastatin in cSDHs which resulted in a decrease of the recurrence after surgical treatment (67). Furthermore, the strong correlation between RPR and cSDH recurrence is also transferrable to an individualized imaging follow-up strategy. RPR might be a useful variable to implement a tailored follow-up strategy and might identify patients who should undergo a more stringent regime of follow-up CT scans despite the highly debated use of routine postoperative CT scans in the absence of persisting deficits or clinical deterioration (68). Moreover, a future trial investigating the use of anti-inflammatory drugs (e.g., dexamethasone or atorvastatin) in the treatment of cSDH might benefit from this biomarker. Increased baseline RPR might be a marker reflecting an increased inflammatory burden in cSDHs patients and facilitate the identification of those patients who can potentially benefit from novel anti-inflammatory approaches.

The present investigation has several limitations. The major limitation is the retrospective design. Furthermore, there are inherent limitations such as preoperative determination of a laboratory value with the risk of increased deviations and the dependency of various potential comorbidities in elderly patients. Moreover, the present study demonstrates that the AUC of RPR in predicting cSDH recurrence was 0.64. The AUC is considered an estimator of overall accuracy. A significant drawback of the AUC in evaluating the diagnostic value is that it reflects a summary of the entire ROC curve, which also might consider areas that are not transferable to practical and clinical use (69). A significant region of the area at the right side of the AUC represents the high false-positive range and might be of minimal clinical value (70). Nevertheless, the present investigation analyzes RPR as a feasible and quick-to-determine prognostic biomarker in patients with cSDH for the first time and might facilitate future trials investigating the inflammatory burden of hematoma fluid and anti-inflammatory treatment methods. Future trials in other neurosurgical institutions will have to provide external validation of this promising correlation between RPR and cSDH recurrence.

## Conclusion

The present investigation demonstrates RPR as a novel marker reflecting inflammatory burden in patients with cSDH in the preoperative risk-benefit assessment and might facilitate a tailored medical decision making regarding therapy and follow-up strategy. Furthermore, RPR might inform the study design of future trials investigating the inflammatory burden in cSDH and facilitate the identification process of patients who might benefit from novel anti-inflammatory therapy approaches in cSDH.

## Data Availability Statement

The raw data supporting the conclusions of this article will be made available by the authors, without undue reservation.

## Ethics Statement

The studies involving human participants were reviewed and approved by Ethic committee of the University of Bonn. Written informed consent for participation was not required for this study in accordance with the national legislation and the institutional requirements.

## Author Contributions

Data acquisition was performed by ÁG and JW. JW, ÁG, M-TS, and EG performed the data interpretation. Writing and creation of figures were performed by JW, ÁG, and EG. Proof reading was done by ÁG, HV, AH, EM, TL, MV, CC, JW, EG, and M-TS.

## Conflict of Interest

The authors declare that the research was conducted in the absence of any commercial or financial relationships that could be construed as a potential conflict of interest.

## Publisher's Note

All claims expressed in this article are solely those of the authors and do not necessarily represent those of their affiliated organizations, or those of the publisher, the editors and the reviewers. Any product that may be evaluated in this article, or claim that may be made by its manufacturer, is not guaranteed or endorsed by the publisher.
